# Bufalin Suppresses Triple-Negative Breast Cancer Stem Cell Growth by Inhibiting the Wnt/β-Catenin Signaling Pathway

**DOI:** 10.4014/jmb.2503.03002

**Published:** 2025-07-18

**Authors:** So Jin Park, Hye Jin Jung

**Affiliations:** 1 Department of Life Science and Biochemical Engineering, Graduate School, Sun Moon University, Asan 31460, Republic of Korea; 2 Department of Pharmaceutical Engineering and Biotechnology, Sun Moon University, Asan 31460, Republic of Korea; 3 Genome-Based BioIT Convergence Institute, Sun Moon University, Asan 31460, Republic of Korea

**Keywords:** Triple-negative breast cancer, cancer stem cell, bufalin, stemness, apoptosis, Wnt/β-catenin

## Abstract

Triple-negative breast cancer (TNBC) is an aggressive subtype of breast cancer with high mortality rates and limited targeted therapies. TNBC stem cells (TNBCSCs) contribute to tumor aggressiveness, metastasis, and treatment resistance. Targeting TNBCSCs represents a promising therapeutic strategy for improving patient outcomes. In this study, we investigated the inhibitory effects of bufadienolides—bufalin, bufotalin, and cinobufotalin—on TNBCSC growth. Among them, bufalin exhibited the strongest antiproliferative activity. We further examined bufalin’s impact on TNBCSC self-renewal, cell cycle regulation, apoptosis, and the Wnt/β-catenin signaling pathway using *in vitro* and *in vivo* models. Bufalin effectively suppressed TNBCSC self-renewal in *in vitro* tumorsphere assays and significantly reduced tumor growth in an *in vivo* HCC1937 TNBCSC xenograft chorioallantoic membrane (CAM) model. Bufalin induced G0/G1 phase cell cycle arrest by downregulating key regulatory proteins, including c-myc, cyclin D1, and CDK4. It also promoted intrinsic apoptosis through nuclear fragmentation, mitochondrial membrane potential reduction, and caspase activation. Additionally, bufalin downregulated key CSC markers, such as CD133, CD44, ALDH1A1, Nanog, Oct4, and Sox2. Notably, bufalin suppressed the Wnt/β-catenin signaling pathway by reducing β-catenin mRNA and protein expression, leading to the downregulation of EGFR, a downstream target of Wnt signaling. Our findings highlight bufalin as a potent chemotherapeutic agent capable of inhibiting TNBCSC growth by targeting stemness, proliferation, and apoptosis through Wnt/β-catenin signaling suppression. These results provide a strong rationale for further investigation of bufalin as a potential therapeutic strategy for TNBC treatment.

## Introduction

Breast cancer is the most frequently diagnosed cancer in women, with around 2.3 million cases and 666,000 related deaths reported worldwide in 2022. It has the highest incidence and mortality rates in several countries [1]. The risk factors for breast cancer include hormonal influences (age, race, menarche, first full-term pregnancy), genetic mutations (BRCA), and lifestyle factors (breastfeeding, alcohol, obesity, environmental hormones) [2].

Triple-negative breast cancer (TNBC) accounts for 15-20% of breast cancer cases and lacks expression of estrogen receptor (ER), progesterone receptor (PR), and human epidermal growth factor receptor 2 (HER2) [3]. It is a highly aggressive tumor with rapidly proliferating cells, a high recurrence rate, and frequent distant metastases, leading to the poorest prognosis among breast cancer subtypes [3]. Standard treatment for TNBC includes surgery, radiation, and chemotherapy, with approved agents such as anthracyclines (*e.g.*, doxorubicin) and taxanes (*e.g.*, paclitaxel) [4]. Additionally, poly (ADP-ribose) polymerase (PARP) inhibitors (*e.g.*, olaparib) and immune checkpoint inhibitors (*e.g.*, atezolizumab) are combined with chemotherapy [5]. However, TNBC’s heterogeneity and complexity often result in drug resistance, recurrence, and treatment failure [6, 7]. Thus, developing more effective therapies for TNBC remains an urgent priority.

Cancer stem cells (CSCs) are a small subset of tumor-initiating cells with self-renewal and pluripotent differentiation capabilities [8]. In TNBC, CSCs (TNBCSCs) drive drug resistance, metastasis, and recurrence [8, 9]. TNBCSCs overexpress key stemness markers such as CD133, CD44, aldehyde dehydrogenase 1A1 (ALDH1A1), Nanog, Oct4, and Sox2, contributing to TNBC’s aggressiveness and poor prognosis [10, 11]. Their self-renewal and differentiation are regulated by CSC-related pathways, including Wnt/β-catenin, Notch, Hedgehog, and JAK/STAT [8, 12]. Targeting these pathways to suppress stemness offers a promising strategy to eliminate TNBCSCs and overcome drug resistance in TNBC.

Apoptosis, a programmed form of cell death, is essential for tissue development and homeostasis [13]. CSCs evade apoptosis by overactivating anti-apoptotic pathways like PI3K/Akt and Wnt/β-catenin and enhancing DNA repair mechanisms [14]. Thus, inducing apoptosis is a key cancer treatment strategy [14]. Apoptosis involves distinct morphological and biochemical changes, including chromatin condensation, DNA fragmentation, reactive oxygen species (ROS) generation, and mitochondrial membrane potential (MMP) loss [13]. It occurs via intrinsic (mitochondria-mediated) or extrinsic (receptor-mediated) pathways, both activating caspases, a family of cysteine proteases [13, 15]. In CSCs, intrinsic pathway dysregulation is driven by overexpression of anti-apoptotic proteins such as Bcl-2, Bcl-xL, and survivin, which inhibit apoptosis [16, 17]. Targeting these proteins to activate caspases may effectively eliminate TNBCSCs.

**Fig. 1:**
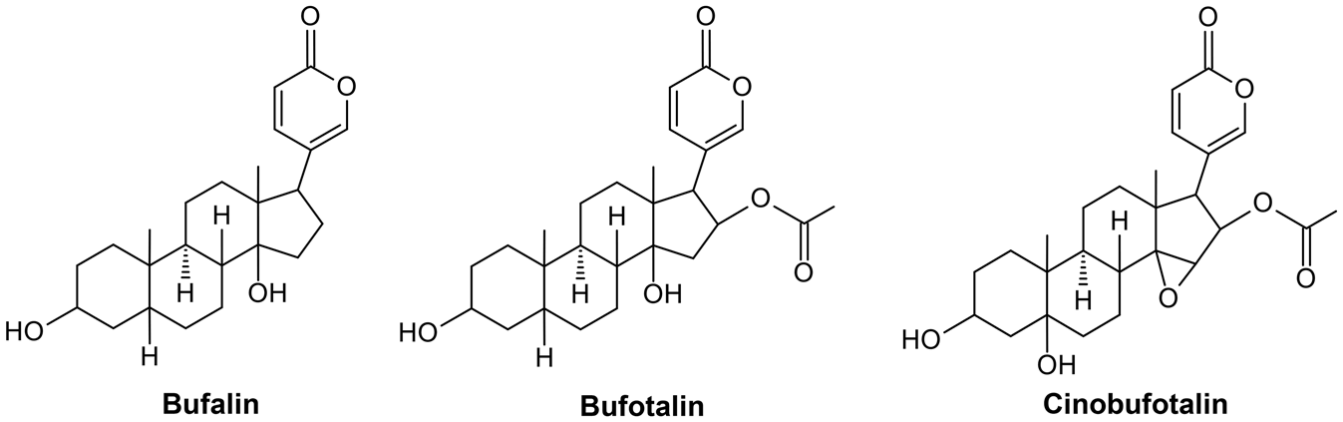
Chemical structures of bufalin, bufotalin, and cinobufotalin.

Bufadienolides are natural cardiac glycosides primarily derived from the skin and parotid gland secretions of *Bufonidae* toads [18]. These C-24 steroids feature a six-membered α-pyrone (lactone) ring at the C-17 position, with over 100 types identified [19]. By interacting with the Na^+^/K^+^-ATPase pump, bufadienolides disrupt ionic homeostasis and modulate signaling pathways, exerting cardiovascular, antimicrobial, anti-inflammatory, and anticancer effects [18]. Among them, bufalin, bufotalin, and cinobufotalin (Fig. 1) show significant anticancer activity in various cancer cell lines [20], but their effects on TNBCSCs remain largely unexplored. This study examines their inhibitory effects on TNBCSCs derived from MDA-MB-231 and HCC1937 cells, focusing on bufalin, which demonstrated the strongest growth suppression, and its role in apoptosis induction and stemness regulation.

## Materials and Methods

### Chemical Reagents

Bufalin, bufotalin, and cinobufotalin were purchased from MedChemExpress (USA). Each compound was dissolved in dimethyl sulfoxide (DMSO) at a concentration of 100 mM to prepare stock solutions and used in the experiments.

### Cell Culture

Human TNBC cell lines MDA-MB-231 (KCLB No. 30026) and HCC1937 (KCLB No. 9S1937) were obtained from the Korea Cell Line Bank (Republic of Korea). Adherent cells were cultured in DMEM and RPMI-1640 (HyClone, USA), respectively, supplemented with 10% fetal bovine serum (FBS) (R&D Systems, USA) and 1%antibiotics (Lonza, USA). Subculturing was performed using trypsin (Gibco, USA). Tumorsphere cells were maintained in serum-free DMEM/F-12 (HyClone) supplemented with 1% antibiotics, 1 × B-27 supplement (Gibco), 20 ng/ml basic fibroblast growth factor (bFGF), 20 ng/ml epidermal growth factor (EGF) (Prospecbio, USA), and 50 ng/ml heparin (Sigma-Aldrich, USA) [21, 22]. Tumorsphere cells were subcultured using accutase (Lonza). All cells were incubated at 37°C with 5% CO_2_ in a humidified incubator (Thermo Fisher Scientific, Finland).

### Cell Viability Assay

TNBCSCs (5 × 10³ cells/well) were seeded in a 96-well white culture plate and treated with bufalin, bufotalin, or cinobufotalin (0-10,000 nM) for 7 days. Cell viability was assessed using the CellTiter-Glo 2.0 Cell Viability Assay (Promega, USA) following the manufacturer's instructions. Luminescence was measured with a microplate reader (BioTek, USA). IC_50_ values were calculated using GraphPad Prism 6 (USA).

### Tumorsphere Formation Assay

TNBCSCs (5 × 10³ cells/well) were seeded in a 96-well culture plate and treated with bufalin, bufotalin, or cinobufotalin (0-625 nM) for 7 days. Tumorspheres (>200 μm) were observed and counted using an optical microscope (Olympus, Japan).

### Chick Embryo Chorioallantoic Membrane (CAM) Assay

The CAM assay is a widely used *in vivo* model, valued for its rapidity, simplicity, and natural immunodeficiency, particularly in studies of angiogenesis, tumorigenesis, toxicology, and drug delivery. Fertilized chicken eggs were incubated at 37°C for 7 days, after which a small window (<1 cm) was made. A mixture of HCC1937-derived TNBCSCs (2 × 10^6^ cells/10 μl), bufalin (2.5 μg/egg), and ECM gel (10 mg/ml, 40 μl/egg) (Sigma-Aldrich) was prepared and solidified in a cell incubator for 1 h. The solidified mixture was then implanted onto the CAM surface and incubated for 10 days. Tumor weight and diameter were measured afterward [21, 22].

### Cell Cycle Analysis

TNBCSCs (2 × 10^5^ cells/well) were plated in a 60-mm culture dish and treated with bufalin (200, 400 nM) for 72 h. Cells were harvested, washed with PBS, and fixed in pre-cooled 70% ethanol at -20°C for 3 h. After fixation, cells were washed with PBS and stained with 200 μl of Muse Cell Cycle reagent (Luminex, USA) following the manufacturer’s protocol. Cell cycle distribution was assessed using the Guava Muse Cell Analyzer with MuseSoft_V1.8.0.3 (Luminex).

### Nucleic Acid Staining

TNBCSCs (1 × 10^5^ cells/well) were seeded in a 24-well culture plate and treated with bufalin (200, 400 nM) for 72 h. Cells were then washed with PBS, stained with 20 μg/ml 4',6-diamidino-2-phenylindole dihydrochloride (DAPI) (Sigma-Aldrich), and incubated for 1 h. Stained nuclei were analyzed using an Optinity KI-2000F fluorescence microscope (Korea Lab Tech, Republic of Korea).

### MMP Measurement

TNBCSCs (1 × 10^5^ cells/well) were seeded in a 24-well culture plate and treated with bufalin (200, 400 nM) for 72 h. Cells were then washed with PBS, stained with 100 nM tetramethylrhodamine ethyl ester (TMRE) (Invitrogen, USA), and incubated for 20 min. MMP was analyzed using a fluorescence microscope, and TMRE fluorescence intensity was quantified using ImageJ 1.5 software (NIH, USA).

### ROS Measurement

TNBCSCs (1 × 10^5^ cells/well) were seeded in 96-black-well culture plates and treated with bufalin (200, 400 nM) for 72 h. Cells were then incubated with 20 μM 2',7'-dichlorodihydrofluorescein diacetate (DCFH-DA) (Sigma-Aldrich) for 30 min. Fluorescence was measured using a microplate reader at an excitation wavelength of 530 nm and an emission wavelength of 590 nm.

### Western Blot Analysis

Cell lysates were prepared with equal protein concentrations and separated by SDS-PAGE. Proteins were transferred onto a PVDF membrane (Cytiva, USA), blocked with 5% skim milk in TBST, and incubated overnight at 4°C with specific primary antibodies (dilution 1:2000 – 1:10,000). The membrane was then incubated with HRP-conjugated secondary antibodies (dilution 1:3000) for 1 h at room temperature, and signals were detected using an enhanced chemiluminescence kit (Dogenbio, Republic of Korea). Antibodies against c-myc (#5605), cyclin D1 (#2922), Bcl-2 (#2872), Bcl-xL (#2764), survivin (#2808), cleaved caspase-3 (#9661), cleaved caspase-9 (#9501), CD133 (#64326), CD44 (#37259), ALDH1A1 (#12035), Nanog (#3580), Oct4 (#2750), Sox2 (#3579), active-β-catenin (#8814), p-β-catenin (#9561), β-catenin (#9562), p-EGFR (#2234), EGFR (#2232), β-actin (#4967), lamin A/C (#2032), mouse IgG (#7076), and rabbit IgG (#7074) were obtained from Cell Signaling Technology (USA). Antibodies against CDK4 (#sc-70831) and Wnt1 (#bs-1739R) were purchased from Santa Cruz Biotechnology (USA) and Bioss (China), respectively. Band intensity was quantified using ImageJ 1.5 software by normalizing the target protein to either β-actin or lamin A/C. The blot images shown are representative of three independent experiments.

### Reverse Transcription Polymerase Chain Reaction (RT-PCR)

Total RNA was extracted using TRIzol reagent (Invitrogen). Complementary DNA (cDNA) was synthesized using M-MLV reverse transcriptase (Bioneer, Republic of Korea) and subsequently amplified with ProFi Taq DNA polymerase (Bioneer) and specific primers for the target genes in a PCR machine (Takara, Japan). PCR products were visualized on a 1.2% agarose gel stained with GreenStar™ Nucleic Acid Staining Solution I (Bioneer). The primers used were: β-Catenin: 5’-ATGACTCGAGCTCAGAGGGT-3’ (F) and 5’-ATTGCACGTGTGGCAAGTTC-3’ (R); GAPDH: 5’-AAGGCTGTGGGCAAGGTCATC-3’ (F) and 5’-GCGTCAAAGGTGGAGGAGTGG-3’ (R). Band intensity was quantified using ImageJ 1.5 software by normalizing the target gene to GAPDH.

### Statistical Analysis

Data are expressed as the mean ± standard deviation (SD). Statistical analysis was performed using ANOVA followed by Tukey’s post-hoc test in SPSS version 9.0 (USA). A *p*-value of < 0.05 was considered statistically significant.

## Results

### Enrichment of TNBCSCs Using Serum-Free Tumorsphere Culture

**Fig. 2:**
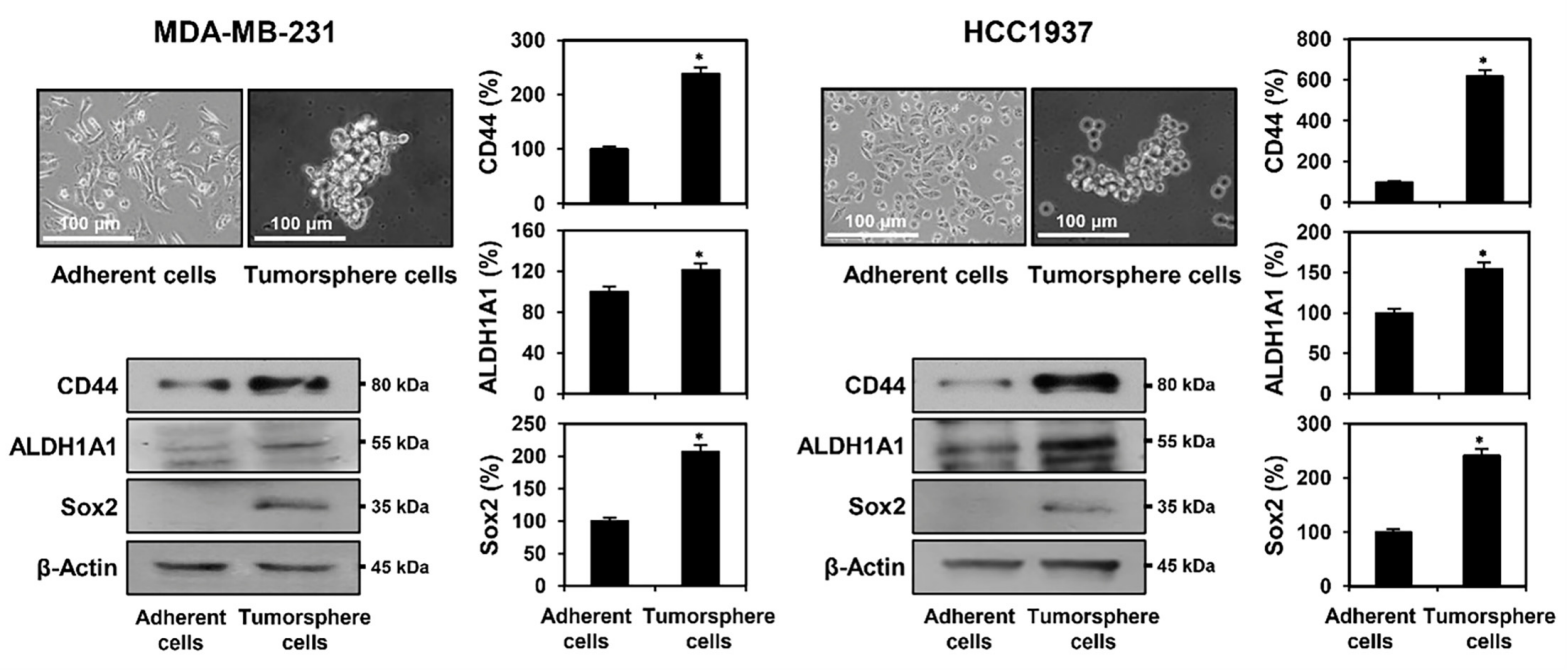
Expression levels of key stemness markers in TNBC adherent and tumorsphere cells. Protein expression levels were analyzed by Western blotting. β-Actin was used as a loading control, and band intensity was quantified by densitometry. *p < 0.05 vs. adherent cells.

The isolation and expansion of CSCs from cancer cells have been successfully achieved through serum-free spheroid suspension culture [23, 24]. To enhance the TNBCSC population, MDA-MB-231 and HCC1937 TNBC cells were cultured in a serum-free tumorsphere system supplemented with EGF and bFGF. The acquisition of stem cell-like characteristics in TNBC cells is linked to the upregulation of stemness-associated factors. Compared to adherent cells maintained in a 10% serum-supplemented medium, tumorsphere-derived MDA-MB-231 and HCC1937 cells exhibited elevated expression of key stemness markers, including CD44, ALDH1A1, and Sox2 (Fig. 2). These findings indicate that serum-free tumorsphere culture effectively enhances the expansion of TNBCSCs in MDA-MB-231 and HCC1937 cells.

### Bufadienolides Suppress TNBCSC Viability

**Fig. 3:**
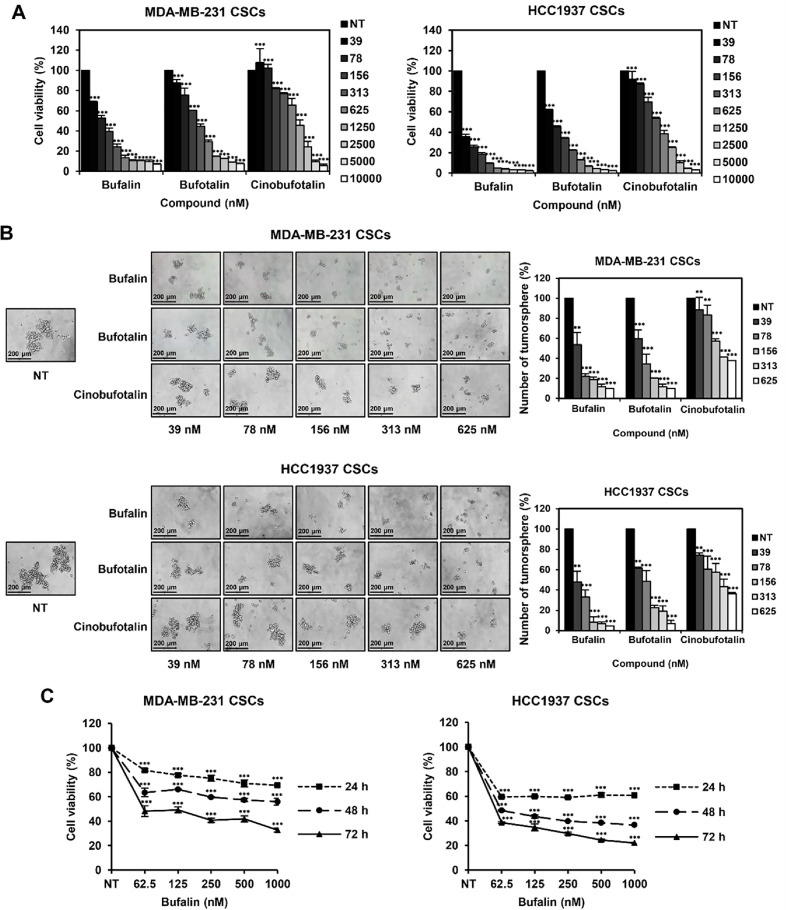
Effects of bufadienolides on TNBCSC viability and tumorsphere formation. (**A**) MDA-MB-231- and HCC1937-derived TNBCSCs were treated with bufalin, bufotalin, or cinobufotalin (0-10,000 nM) for 7 days, and cell viability was assessed using the CellTiter-Glo^®^ luminescent assay. (**B**) TNBCSCs were treated with bufalin, bufotalin, or cinobufotalin (0-625 nM) for 7 days, and tumorsphere formation was observed and quantified. (**C**) TNBCSCs were treated with bufalin (0-1000 nM) for 24, 48, and 72 h, and cell viability was measured using the CellTiter-Glo^®^ luminescent assay. **p < 0.01, ***p < 0.001 vs. control.

The effect of bufadienolides on TNBCSC viability was assessed using a luminescent ATP detection assay. MDA-MB-231- and HCC1937-derived TNBCSCs were treated with bufalin, bufotalin, and cinobufotalin (0 – 10,000 nM) for 7 days. Bufalin exhibited the strongest inhibitory effect, with IC_50_ values of 91 nM and 18 nM in MDA-MB-231-and HCC1937-derived TNBCSCs, respectively (Fig. 3A). The IC_50_ values for bufotalin were 255 nM and 67 nM, while cinobufotalin showed the weakest effect, with IC_50_ values of 900 nM and 371 nM, respectively (Fig. 3A).

To examine their impact on tumorsphere formation, TNBCSCs were treated with bufalin, bufotalin, and cinobufotalin (0–625 nM) for 7 days, and tumorsphere counts were analyzed. Among the three compounds, bufalin most significantly reduced both the number and size of tumorspheres (Fig. 3B), highlighting its superior inhibitory effect on TNBCSC growth. Based on these findings, further investigations focused on bufalin.

To determine its time-dependent effect on cell viability, TNBCSCs were treated with bufalin (0–1,000 nM) for 24, 48, and 72 h. The results confirmed that bufalin suppressed TNBCSC viability in a time-dependent manner (Fig. 3C). Collectively, these findings demonstrate that bufalin exerts the most potent inhibitory effect on TNBCSC growth in a dose- and time-dependent manner.

We also compared the cytotoxic effects of bufalin between TNBC and normal cell lines. The 267B1 normal prostate epithelial cell line was used to evaluate bufalin’s cytotoxicity in non-cancerous cells. Although not tissue-matched to breast cancer, it served as a general indicator of normal cell sensitivity due to the unavailability of normal human mammary epithelial cells. Bufalin more effectively reduced the viability of MDA-MB-231 and HCC1937 TNBC cells compared to 267B1 normal prostate cells (Fig. S1). These results suggest that bufalin exerts stronger cytotoxic effects on TNBC cells than on normal cells.

### Bufalin Suppresses *In Vivo* Tumor Growth in HCC1937-Derived TNBCSCs

**Fig. 4:**
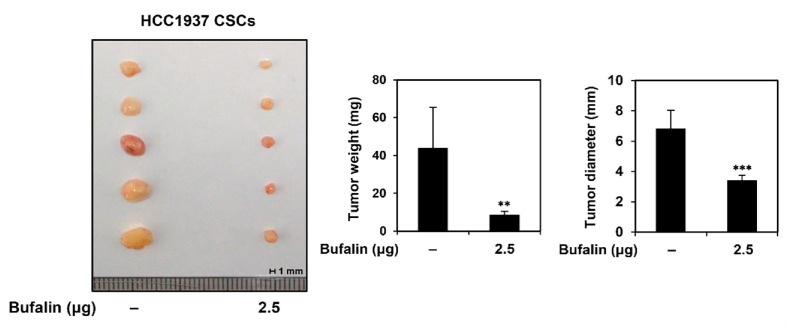
Effect of bufalin on *in vivo* tumorigenesis of HCC1937-derived TNBCSCs. HCC1937-derived TNBCSCs were mixed with ECM gel with or without bufalin (2.5 μg/egg) and implanted onto the CAM of fertilized chick eggs. After 10 days of incubation, tumors were collected, and tumor weight (control: 43.8 ± 21.6 mg, bufalin: 8.4 ± 2.1 mg) and diameter (control: 6.8 ± 1.2 mm, bufalin: 3.4 ± 0.4 mm) were measured. **p < 0.01, ***p < 0.001 vs. control group.

To assess the effect of bufalin on the tumorigenic potential of TNBCSCs *in vivo*, a CAM model was utilized. HCC1937-derived TNBCSCs were mixed with ECM gel and 2.5 μg of bufalin, then injected onto the CAM surface and incubated for 10 days. Tumor weight and diameter were subsequently measured. In the control group, the tumor weight was 43.8 ± 21.6 mg, with a diameter of 6.8 ± 1.2 mm (Fig. 4). In contrast, bufalin treatment significantly reduced tumor size, with a weight of 8.4 ± 2.1 mg and a diameter of 3.4 ± 0.4 mm. These findings indicate that bufalin possesses strong therapeutic potential in suppressing the tumorigenic capacity of TNBCSCs *in vivo*.

### Bufalin Induces G0/G1 Phase Cell Cycle Arrest in TNBCSCs

**Fig. 5:**
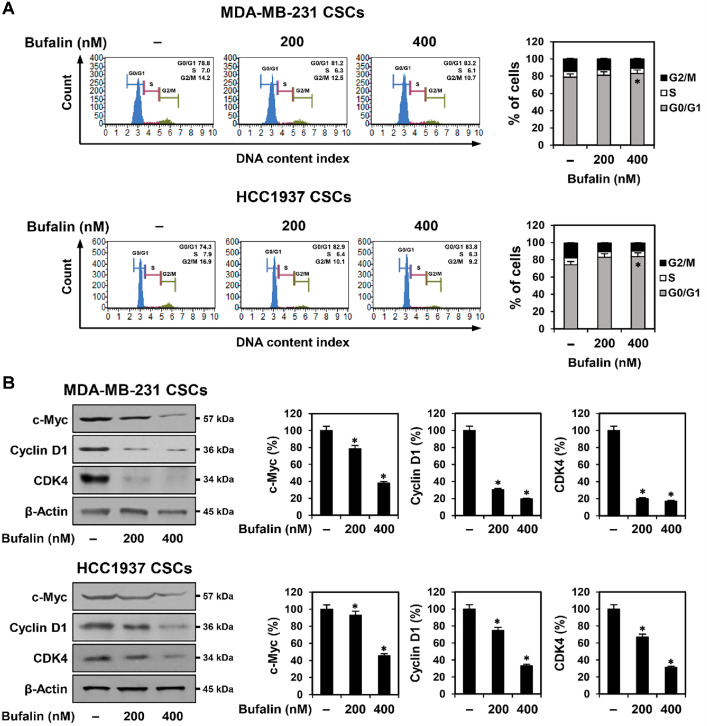
Effect of bufalin on the cell cycle of TNBCSCs. MDA-MB-231- and HCC1937-derived TNBCSCs were treated with bufalin (200, 400 nM) for 72 h. (**A**) Cell cycle distribution was analyzed using the Muse^®^ Cell Analyzer after staining with the Muse^®^ Cell Cycle reagent. (**B**) Protein expression levels of cell cycle-related proteins were assessed by Western blotting. β- Actin was used as a loading control, and band intensity was quantified by densitometry. * *p* < 0.05 vs. control.

To investigate whether bufalin’s inhibitory effect on TNBCSC growth is linked to cell cycle regulation, flow cytometry analysis was conducted. MDA-MB-231- and HCC1937-derived TNBCSCs were treated with bufalin (200, 400 nM) for 72 h. Compared to the control, bufalin increased the proportion of cells in the G0/G1 phase while reducing the S and G2/M phase populations in both cell lines (Fig. 5A). These findings indicate that bufalin suppresses TNBCSC growth by inducing G0/G1 phase cell cycle arrest.

c-Myc is a key transcription factor that regulates G1 phase progression, while cyclin D1 and CDK4 are crucial checkpoint proteins that facilitate the transition from G1 to the S phase [25, 26]. To determine whether bufalin affects the expression of these regulators, Western blot analysis was performed. After 72 h of bufalin treatment, the expression levels of c-myc, cyclin D1, and CDK4 were reduced in both MDA-MB-231- and HCC1937-derived TNBCSCs (Fig. 5B). These results suggest that bufalin-induced G0/G1 phase arrest is associated with the downregulation of key cell cycle regulators, ultimately contributing to TNBCSC growth inhibition.

### Bufalin Activates the Intrinsic Apoptotic Pathway in TNBCSCs

**Fig. 6:**
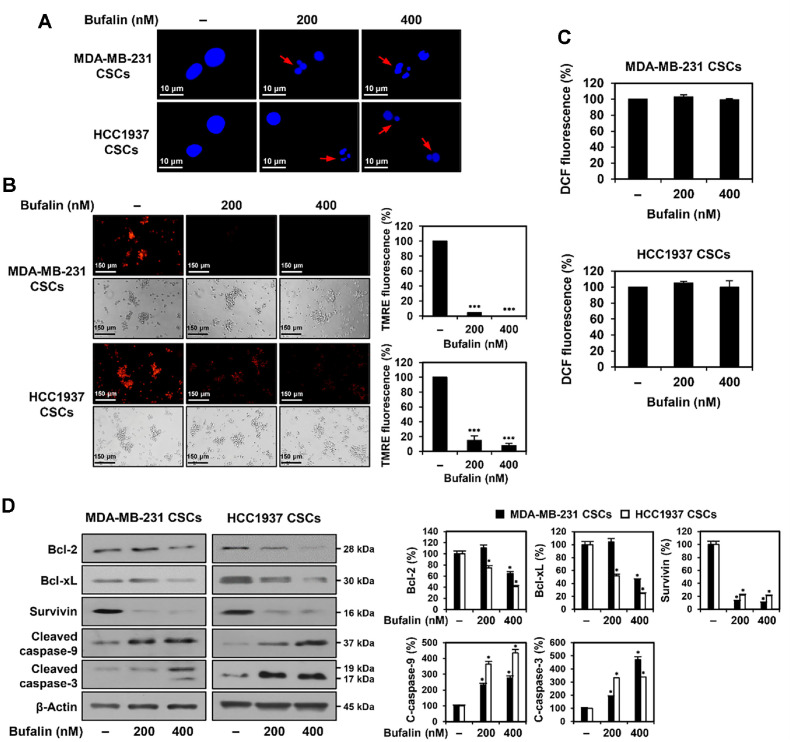
Effect of bufalin on the intrinsic apoptotic pathway in TNBCSCs. MDA-MB-231- and HCC1937-derived TNBCSCs were treated with bufalin (200, 400 nM) for 72 h. (**A**) Nuclear morphology was analyzed by DAPI staining under a fluorescence microscope. Condensed and fragmented nuclei are indicated by red arrows. (**B**) MMP was assessed by TMRE staining and quantified by densitometry. (**C**) Intracellular ROS levels were measured using DCFH-DA staining, and DCF fluorescence was quantified with a microplate reader. (**D**) Protein expression levels of apoptosis regulators were analyzed by Western blotting. β-Actin was used as a loading control, and band intensity was quantified by densitometry. * *p* < 0.05, *** *p* < 0.001vs. control.

To investigate whether bufalin induces apoptosis in TNBCSCs, we analyzed nuclear morphological changes, MMP, and ROS levels, which are key indicators of mitochondria-mediated apoptosis [13]. Following 72 h of bufalin treatment, DAPI staining revealed nuclear condensation and fragmentation in MDA-MB-231- and HCC1937-derived TNBCSCs, compared to the control group (Fig. 6A). MMP was assessed using TMRE, a red-orange cationic fluorescent dye, showing a significant reduction in MMP in both cell lines after bufalin treatment (Fig. 6B). Additionally, intracellular ROS levels were measured using the redox-sensitive fluorescent probe DCFH-DA; however, no significant change was observed after 72 h of bufalin treatment compared to the control (Fig. 6C). These findings suggest that bufalin induces apoptosis through a mitochondria-mediated, ROS-independent pathway.

To further investigate the molecular mechanism of bufalin-induced apoptosis, Western blot analysis was performed to assess the expression of apoptosis-related proteins. Bcl-2 and Bcl-xL, known anti-apoptotic proteins, prevent apoptosis by inhibiting mitochondrial apoptotic signaling, while survivin, a member of the inhibitor of apoptosis protein (IAP) family, plays a crucial role in suppressing the intrinsic caspase-9-dependent apoptotic pathway [16, 17]. After 72 h of bufalin treatment, the expression levels of Bcl-2, Bcl-xL, and survivin were downregulated, whereas cleaved caspase-9 and caspase-3, key pro-apoptotic proteins, were upregulated in both MDA-MB-231- and HCC1937-derived TNBCSCs (Fig. 6D). These results demonstrate that bufalin induces apoptosis in TNBCSCs by activating the intrinsic apoptotic pathway.

### Bufalin Downregulates Stemness Markers in TNBCSCs

**Fig. 7:**
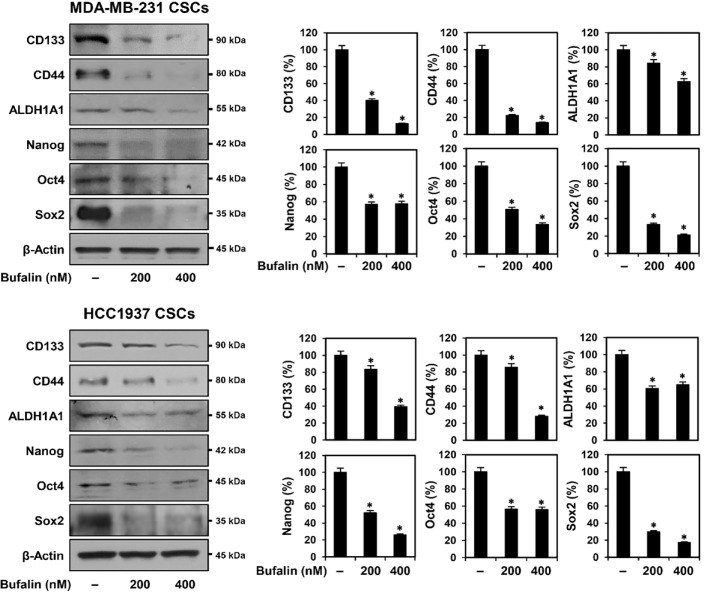
Effect of bufalin on stemness marker expression in TNBCSCs. MDA-MB-231- and HCC1937-derived TNBCSCs were treated with bufalin (200, 400 nM) for 72 h. Protein expression levels of stemness markers were analyzed by Western blotting. β-Actin was used as a loading control, and band intensity was quantified by densitometry. * *p* < 0.05 vs. control.

To investigate whether bufalin affects stemness markers in TNBCSCs, Western blot analysis was conducted. The cell surface markers CD133 and CD44 are critical for identifying TNBCSCs and maintaining their tumor-initiating capacity, facilitating self-renewal and metastasis [10]. Additionally, the cytosolic detoxifying enzyme ALDH1A1 and the intracellular transcription factors Nanog, Oct4, and Sox2 play key roles in regulating stemness, cell differentiation, and drug resistance, with their elevated expression contributing to TNBCSC survival and aggressiveness [10, 11]. Following 72 h of bufalin treatment, the expression levels of CD133, CD44, ALDH1A1, Nanog, Oct4, and Sox2 were significantly reduced in both MDA-MB-231- and HCC1937-derived TNBCSCs (Fig. 7). These findings suggest that bufalin exerts its anticancer effects by suppressing key stemness markers in TNBCSCs.

### Bufalin Inhibits the Wnt/β-Catenin Signaling Pathway in TNBCSCs

**Fig. 8:**
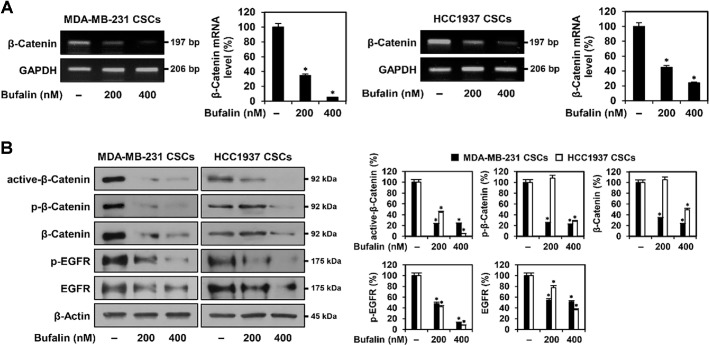
Effect of bufalin on the Wnt/β-catenin signaling pathway in TNBCSCs. MDA-MB-231- and HCC1937- derived TNBCSCs were treated with bufalin (200, 400 nM) for 72 h. (**A**) Total RNA was extracted, and cDNA was synthesized via reverse transcription. β-Catenin mRNA levels were analyzed by RT-PCR. GAPDH served as an internal control, and band intensity was quantified by densitometry. (**B**) Protein expression levels of β-catenin and EGFR were assessed by Western blotting. β-Actin was used as a loading control, and band intensity was quantified by densitometry. * *p* < 0.05 vs. control.

To evaluate whether bufalin affects β-catenin transcription, a key regulator of the Wnt/β-catenin signaling pathway involved in stem cell maintenance, proliferation, and survival in TNBCSCs [27], RT-PCR analysis was performed. After 72 h of bufalin treatment, β-catenin mRNA levels were significantly reduced in both MDA-MB-231- and HCC1937-derived TNBCSCs (Fig. 8A). These findings suggest that bufalin may suppress TNBCSC growth by inhibiting β-catenin transcription.

To determine whether this transcriptional downregulation also leads to reduced β-catenin protein levels, Western blot analysis was conducted. β-Catenin exists in both active (non-phosphorylated) and inactive (phosphorylated) states, depending on Wnt signaling activity [28]. In the presence of Wnt signaling, active β-catenin translocates to the nucleus, where it interacts with TCF/LEF transcription factors to regulate downstream genes, including *c-myc*, *cyclin D1*, and *EGFR*, promoting cell proliferation. In contrast, in the absence of Wnt signaling, β-catenin is phosphorylated at Ser33/37 and Thr41 by GSK3β, leading to ubiquitination and proteasomal degradation, thereby preventing its activation. Total β-catenin represents the combined levels of both active and inactive forms. Following 72 h of bufalin treatment, the expression levels of all forms of β-catenin (active, inactive, and total), as well as phosphorylated and total EGFR, were decreased in both MDA-MB-231- and HCC1937-derived TNBCSCs (Fig. 8B). Bufalin also reduced β-catenin protein levels in both the cytoplasmic and nuclear fractions of TNBCSCs (Fig. S2). In contrast, Wnt1 protein levels, the upstream activator of β-catenin, showed a slight increase following bufalin treatment, suggesting that bufalin does not directly inhibit Wnt1 expression (Fig. S3). These findings indicate that bufalin exerts its anticancer effects in TNBCSCs by specifically downregulating the Wnt/β-catenin signaling pathway at the level of β-catenin.

## Discussion

TNBC is an aggressive breast cancer subtype with a high mortality rate and a lack of effective targeted therapies [1, 3]. TNBCSCs play a key role in TNBC’s aggressiveness and treatment resistance, driving metastasis and contributing to high recurrence rates and poor patient survival [3]. Therefore, targeting TNBCSCs presents a promising strategy for improving patient outcomes and prognosis in TNBC.

Bufadienolides are a class of cardiotonic steroids characterized by a steroid backbone and a six-membered unsaturated lactone ring at C17 [18]. They are primarily found in toad venom and exhibit cardiotonic, anti-inflammatory, and anticancer properties [18]. Their biological activity is mainly attributed to their ability to inhibit Na^+^/K^+^-ATPase, which triggers downstream cellular effects [18].

In this study, we first evaluated the inhibitory effects of bufadienolides—specifically bufalin, bufotalin, and cinobufotalin—on the growth of TNBCSCs. Among them, bufalin demonstrated the strongest antiproliferative activity. While bufalin, bufotalin, and cinobufotalin share a similar bufadienolide structure, they differ in their functional groups [18, 19]. Bufalin’s structural features may enable more effective binding to cellular targets such as Na^+^/K^+^-ATPase, leading to enhanced downstream signaling that suppresses TNBCSC growth.

Bufalin has exhibited potent anticancer effects across various cancer types by inhibiting cell growth, inducing cell cycle arrest, and promoting apoptosis [29]. Its ability to target CSCs has also been increasingly recognized in multiple malignancies. In colorectal cancer cells, bufalin reversed cisplatin-induced stemness and drug resistance by downregulating stemness-related markers [30]. In gemcitabine-resistant pancreatic cancer cells, it reduced the CSC population by modulating the Hedgehog signaling pathway [31]. Similarly, bufalin suppressed the proliferation and stemness of osteosarcoma CSCs by upregulating miR-148a expression [32]. In glioblastoma stem cells, it enhanced sensitivity to temozolomide by activating the mitochondrial apoptotic pathway [33]. Additionally, bufalin downregulated key stemness-associated transcription factors, Sox2 and Oct4, in TNBC cells [34]. These findings highlight bufalin’s strong potential as a natural therapeutic agent for eradicating CSCs. However, the precise molecular mechanisms underlying its suppression of TNBCSC growth remain incompletely understood.

**Fig. 9:**
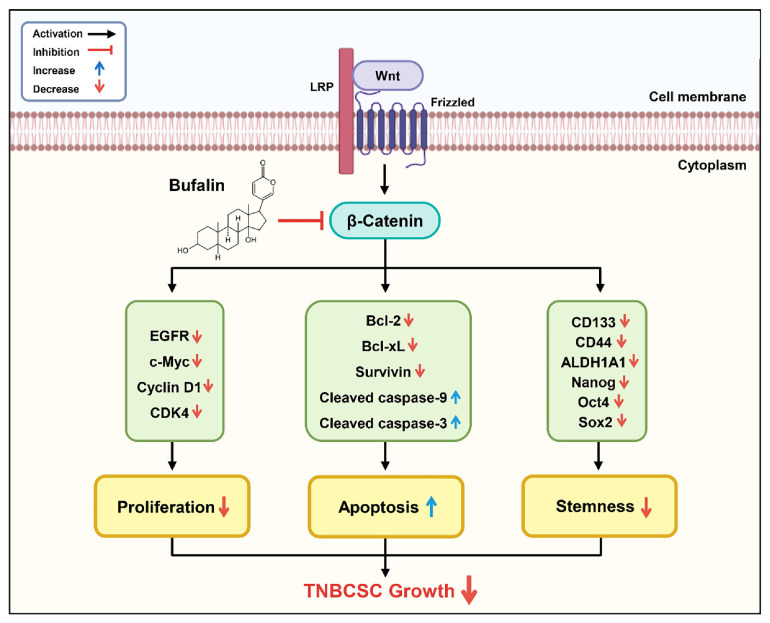
The molecular regulatory mechanisms by which bufalin suppresses TNBCSC growth.

Our study demonstrated that bufalin effectively suppressed *in vitro* self-renewal capacity in MDA-MB-231- and HCC1937-derived TNBCSCs and reduced *in vivo* tumor growth in the HCC1937 TNBCSC xenograft CAM model. Bufalin induced G0/G1 phase cell cycle arrest by downregulating key regulatory factors, including c-myc, cyclin D1, and CDK4, in both TNBCSCs. Additionally, bufalin activated critical apoptotic mechanisms, such as nuclear fragmentation, MMP reduction, and caspase cascade activation, leading to intrinsic apoptosis in TNBCSCs. Furthermore, bufalin downregulated key CSC markers, including CD133, CD44, ALDH1A1, Nanog, Oct4, and Sox2. Notably, the growth-suppressive effect of bufalin was linked to the inhibition of β-catenin, a key regulator of the Wnt/β-catenin signaling pathway. Bufalin reduced both mRNA and protein levels of β-catenin, subsequently downregulating key Wnt target genes, such as *EGFR*, *c-myc*, and *cyclin D1*. These findings suggest that bufalin possesses strong chemotherapeutic potential for effectively inhibiting TNBCSC growth (Fig. 9).

The Wnt/β-catenin signaling pathway plays a critical role in maintaining CSCs by regulating their self-renewal, proliferation, and survival [35-37]. Dysregulation of this pathway can lead to the aberrant activation of genes that enhance CSC properties, making tumors more aggressive and resistant to treatment. The Wnt/β-catenin pathway promotes the expression of key stemness markers such as Nanog, Oct4, Sox2, and ALDH1A1, which are essential for sustaining CSC self-renewal [38, 39]. Additionally, it upregulates proliferation-associated markers, including c-myc, cyclin D1, EGFR, and LGR5, contributing to CSC proliferation and differentiation [27]. Moreover, the Wnt/β-catenin pathway enhances CSC survival by activating anti-apoptotic signals that prevent programmed cell death. It increases the production of proteins such as Bcl-2 and survivin, which enable CSCs to evade apoptosis [14, 40]. Given its role in maintaining CSC properties, Wnt/β-catenin pathway activation is associated with increased treatment resistance and tumor progression. Compared to non-TNBCs, TNBC exhibits a higher abundance of Wnt pathway-related genes [35]. Aberrant Wnt/β-catenin signaling has been linked to enhanced stemness and chemotherapy resistance in TNBC [35].

Our findings demonstrate that bufalin suppresses TNBCSC growth by inhibiting stemness and proliferation while promoting apoptosis through the downregulation of β-catenin expression, a key regulator of the Wnt/β-catenin signaling pathway. In conclusion, this study highlights bufalin as a promising anticancer agent for effectively targeting TNBCSCs. Although the *in vivo* efficacy of bufalin was evaluated using the CAM assay, additional *in vivo* studies using tumor xenograft mouse models are necessary to conclusively validate its effectiveness and safety in suppressing TNBCSC-derived tumor growth. Moreover, future studies incorporating normal breast epithelial cells will be important to further confirm the clinical relevance and tissue-specific selectivity of bufalin’s anticancer effects.

## Supplemental Materials

Supplementary data for this paper are available on-line only at http://jmb.or.kr.
