# Supplementary material for: Bufalin Suppresses Triple-Negative Breast Cancer Stem Cell Growth by Inhibiting the Wnt/β-Catenin Signaling Pathway
Source: J Microbiol Biotechnol. 2025 Jul 18;35:e2503002. doi: 10.4014/jmb.2503.03002 (PMC12324995; doi:10.4014/jmb.2503.03002)
Supplement: Supplementary file 1 [file jmb-35-e2503002-supple.pdf]

**J. Microbiol. Biotechnol. 2025. 35: e2503002**

**https://doi.org/10.4014/jmb.2503.03002**

# Supplementary Figures

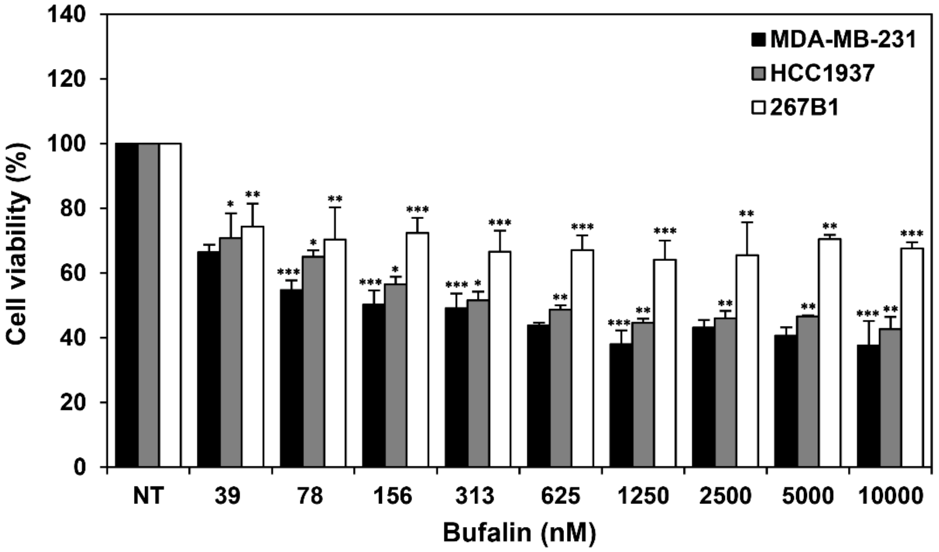


**Fig. S1. Comparison of bufalin-induced cytotoxicity in TNBC and normal cell lines.** TNBC (MDA-MB-231, HCC1937) and normal prostate (267B1) cells were treated with bufalin (0–10,000 nM) for 24 h, and cell viability was measured using the MTT assay. *$p < 0.05$, **$p < 0.01$, ***$p < 0.001$ vs. control.

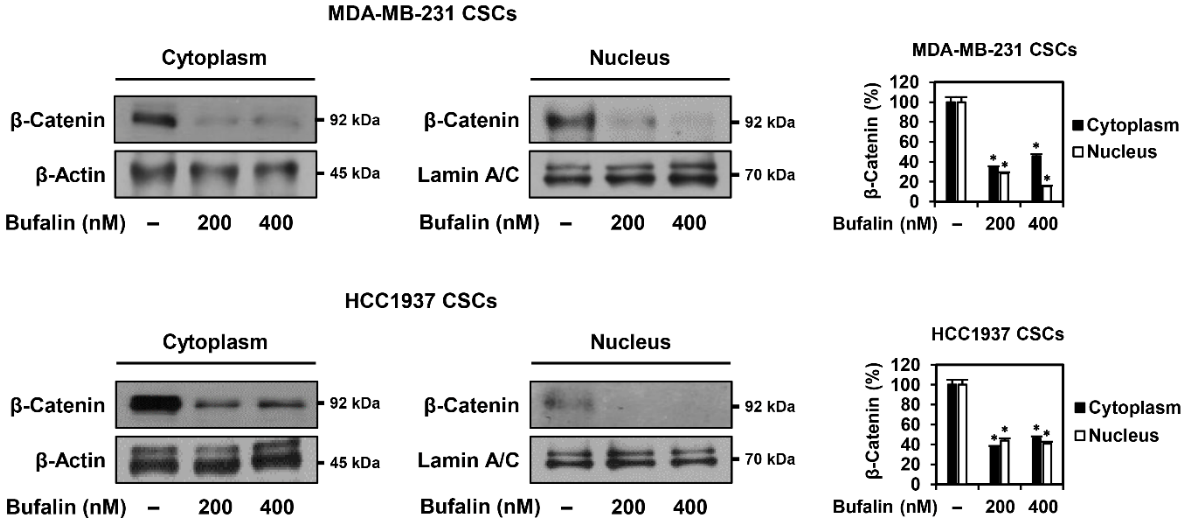


**Fig. S2. Bufalin reduces β-catenin protein levels in both the cytoplasmic and nuclear fractions of TNBCSCs.** MDA-MB-231- and HCC1937-derived TNBCSCs were treated with bufalin (200 or 400 nM) for 72 h. After cell harvesting, cytoplasmic and nuclear fractions were prepared. β-Catenin protein levels were analyzed by Western blotting. β-Actin and lamin A/C were used as loading controls for cytoplasmic and nuclear proteins, respectively. Band intensities were quantified by densitometry. *$p < 0.05$ vs. control.

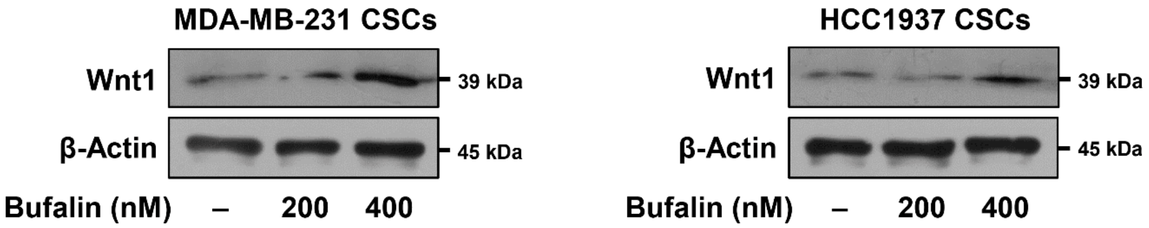


**Fig. S3. Effect of bufalin on Wnt1 expression in TNBCSCs.** MDA-MB-231- and HCC1937-derived TNBCSCs were treated with bufalin (200, 400 nM) for 72 h. Protein expression levels of Wnt1 were analyzed by Western blotting. β-Actin was used as a loading control.
